# Supplementary material for: Accounting for motion in resting-state fMRI: What part of the spectrum are we characterizing in autism spectrum disorder?
Source: Neuroimage. Author manuscript; Available in PMC 2022 Aug 15. (PMC9233079; doi:10.1016/j.neuroimage.2022.119296)
Supplement: web supplement [file NIHMS1805367-supplement-web_supplement.pdf]

Web Supplement to: Accounting for motion in resting-state fMRI: What part of the spectrum are we characterizing in autism spectrum disorder?

Mary Beth Nebel[a,b,*], Daniel E. Lidstone[a,b], Liwei Wang[c], David Benkeser[c], Stewart H. Mostofsky[a,b,d], Benjamin B. Risk[c]

[a] *Center for Neurodevelopmental and Imaging Research, Kennedy Krieger Institute*
[b] *Department of Neurology, Johns Hopkins University School of Medicine*
[c] *Department of Biostatistics and Bioinformatics, Emory University*
[d] *Department of Psychiatry and Behavioral Science, Johns Hopkins University School of Medicine*

Table S.1: **Summary of Mann-Whitney U tests comparing included and excluded participants using the lenient motion QC and stratified by primary diagnosis.** Medians for included and excluded participants are reported, as well as effect sizes (r) and FDR-adjusted p values. ASD=autism spectrum disorder. ADOS=Autism Diagnostic Observation Schedule total score. GAI=General Ability Index. SRS=Social Responsiveness Scale. TD=typically developing

Primary Diagnosis	Variable	Included	Excluded	r	p
ASD	ADOS	13.00	16.00	0.16	0.07
ASD	SRS	91.25	100.00	0.19	0.05
ASD	Motor Overflow	17.00	22.00	0.27	0.01
ASD	Inattention	18.00	16.00	0.01	0.75
ASD	Hyperactivity	11.00	12.00	0.03	0.42
ASD	Age	10.54	9.76	0.14	0.08
ASD	GAI	108.00	101.00	0.15	0.07
TD	SRS	16.00	15.00	<0.01	0.54
TD	Motor Overflow	11.00	13.00	0.08	0.08
TD	Inattention	2.00	2.00	0.03	0.34
TD	Hyperactivity	1.00	2.00	0.11	0.07
TD	Age	10.32	9.87	0.12	0.05
TD	GAI	116.00	112.50	0.10	0.07

*716 N Broadway, Baltimore, MD 21205
Email address: mb@jhmi.edu (Mary Beth Nebel)

Table S.2: **Summary of Mann-Whitney U tests comparing included and excluded participants using the strict motion QC and stratified by primary diagnosis.** Medians for included and excluded participants are reported, as well as effect sizes (r) and FDR-adjusted p values. ASD=autism spectrum disorder. ADOS=Autism Diagnostic Observation Schedule total score. GAI=General Ability Index. SRS=Social Responsiveness Scale. TD=typically developing

Primary Diagnosis	variable	Included	Excluded	r	p
ASD	ADOS	12.50	14.00	0.17	0.08
ASD	SRS	84.25	93.50	0.17	0.08
ASD	Motor Overflow	15.00	18.00	0.15	0.10
ASD	Inattention	15.50	18.00	0.06	0.25
ASD	Hyperactivity	11.00	12.00	0.07	0.25
ASD	Age	11.04	10.13	0.20	0.05
ASD	GAI	107.50	107.00	0.02	0.40
TD	SRS	14.25	17.00	0.10	0.10
TD	Motor Overflow	11.00	12.00	0.06	0.20
TD	Inattention	2.00	2.00	0.05	0.20
TD	Hyperactivity	1.00	2.00	0.16	0.02
TD	Age	10.38	10.13	0.09	0.10
TD	GAI	116.00	115.00	0.07	0.14

Table S.3: **Summary of edges for which DRTMLE indicated a group difference at FDR=.20**. Naïve group mean functional connectivity estimates, naïve Z-Statistics for ASD-TD, DRTMLE group mean functional connectivity estimates, DRTMLE Z-Statistics for ASD-TD, naïve FDR-adjusted p-values, and DRTMLE FDR-adjusted p-values are displayed. Mean functional connectivity group estimates further from zero reflect stronger functional connectivity regardless of sign. Positive mean functional connectivity estimate reflect positive partial correlations, or more integrated intrinsic activity between independent components (ICs). Negative mean functional connectivity estimates reflect negative partial correlations or more segregated intrinsic activity between ICs. Edges are ordered by naïve FDR-adjusted p-values. ASD=autism spectrum disorder. TD=typically developing. Attn=dorsal attention. CB=pontomedullary/cerebellar. Control=executive control. Default=default mode. SalVenAtt=salience/ventral attention. SomMot=somatomotor. Vis=visual.

Edge	Network Pair	naive ASD mean	naive TD mean	naive ASD-TD Z	DRTMLE ASD mean	DRTMLE TD mean	DRTMLE ASD-TD Z	naive FDR-adjusted p	DRTMLE FDR-adjusted p
2-27	Vis-Control	-0.038	-0.024	-4.088	-0.040	-0.024	-4.67	0.007	0.000
14-19	CB-Attn	0.068	0.078	-3.351	0.068	0.078	-3.18	0.062	0.045
13-26	SomMot-Default	-0.031	-0.039	2.805	-0.030	-0.039	3.12	0.128	0.046
14-21	CB-Control	0.044	0.053	-2.862	0.041	0.052	-3.36	0.128	0.045
19-25	Attn-Default	-0.085	-0.094	2.867	-0.086	-0.094	2.45	0.128	0.168
19-26	Attn-Default	0.037	0.028	2.997	0.037	0.027	3.29	0.128	0.045
4-17	CB-Default	-0.067	-0.076	2.671	-0.067	-0.076	3.19	0.165	0.045
17-24	Default-Control	-0.005	0.003	-2.561	-0.006	0.003	-2.94	0.200	0.063
1-14	SomMot-CB	-0.002	0.007	-2.225	-0.003	0.006	-2.41	0.399	0.168
14-24	CB-Control	0.015	0.021	-2.099	0.014	0.021	-2.30	0.457	0.168
21-30	Control-SalVenAtt	0.063	0.069	-2.115	0.063	0.069	-2.33	0.457	0.168
13-25	SomMot-Default	-0.097	-0.102	1.869	-0.094	-0.102	2.66	0.470	0.109
15-30	SomMot-SalVenAtt	0.113	0.117	-1.830	0.112	0.117	-2.29	0.470	0.168
19-24	Attn-Control	-0.160	-0.165	2.037	-0.159	-0.165	2.25	0.470	0.171
4-21	CB-Control	-0.041	-0.034	-1.918	-0.043	-0.034	-2.17	0.470	0.184
8-22	Vis-SalVenAtt	0.019	0.024	-1.782	0.017	0.025	-2.48	0.470	0.168
26-30	Default-SalVenAtt	-0.088	-0.082	-1.720	-0.090	-0.082	-2.29	0.503	0.168
1-24	SomMot-Control	-0.027	-0.032	1.695	-0.023	-0.033	2.74	0.510	0.094
1-21	SomMot-Control	0.003	-0.002	1.668	0.008	-0.002	3.02	0.521	0.055
13-22	SomMot-SalVenAtt	-0.030	-0.035	1.564	-0.029	-0.035	2.35	0.522	0.168
17-27	Default-Control	0.034	0.028	1.557	0.036	0.028	2.17	0.522	0.184
13-24	SomMot-Control	-0.091	-0.095	1.117	-0.088	-0.095	2.24	0.679	0.171
15-21	SomMot-Control	0.039	0.042	-1.098	0.038	0.044	-2.23	0.679	0.171
19-21	Attn-Control	-0.009	-0.013	1.086	-0.006	-0.015	2.75	0.679	0.094
17-25	Default-Default	0.033	0.032	0.425	0.041	0.031	2.31	0.916	0.168

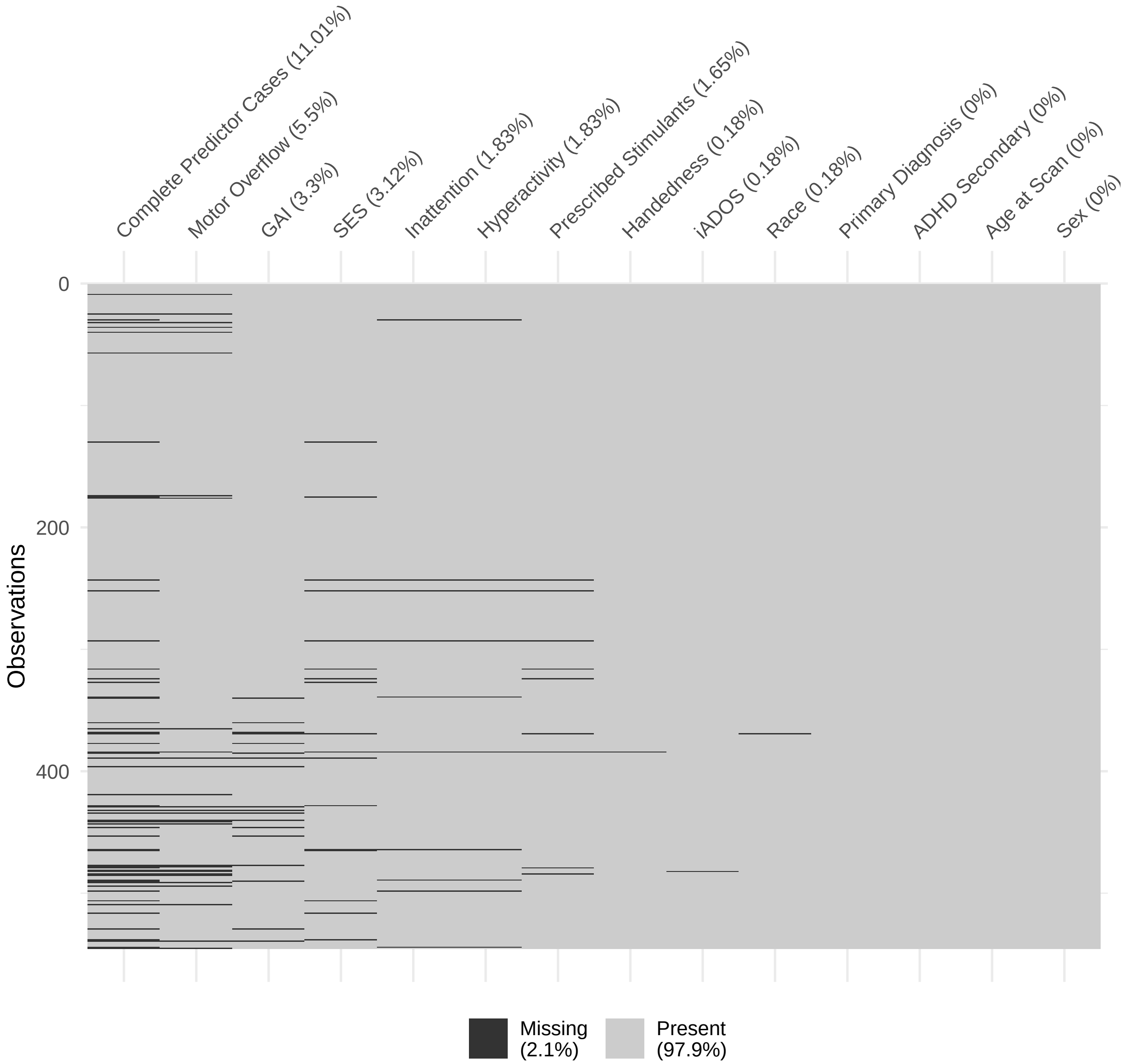

Figure S.1: **Missingness of socio-demographic and behavioral variables in the initial KKI dataset.** There are 545 children in the aggregated KKI dataset. The sample used in DRTMLE is defined by the observations containing data for the variables in the propensity and outcome models together with the socio-demographic variables used in adjusted residuals (i.e., Socioeconomic Status [SES], Sex, and Race). Ordered by the percent missingness, the variables used to define the complete predictor cases are GAI, SES, Motor Overflow, Inattention, Hyperactivity, Prescribed Stimulants, Handedness, Autism Diagnostic Observation Schedule total score including assignment ADOS=0 for typically developing (iADOS), Race, Primary Diagnosis, ADHD Secondary Diagnosis, Age at Scan, and Sex.

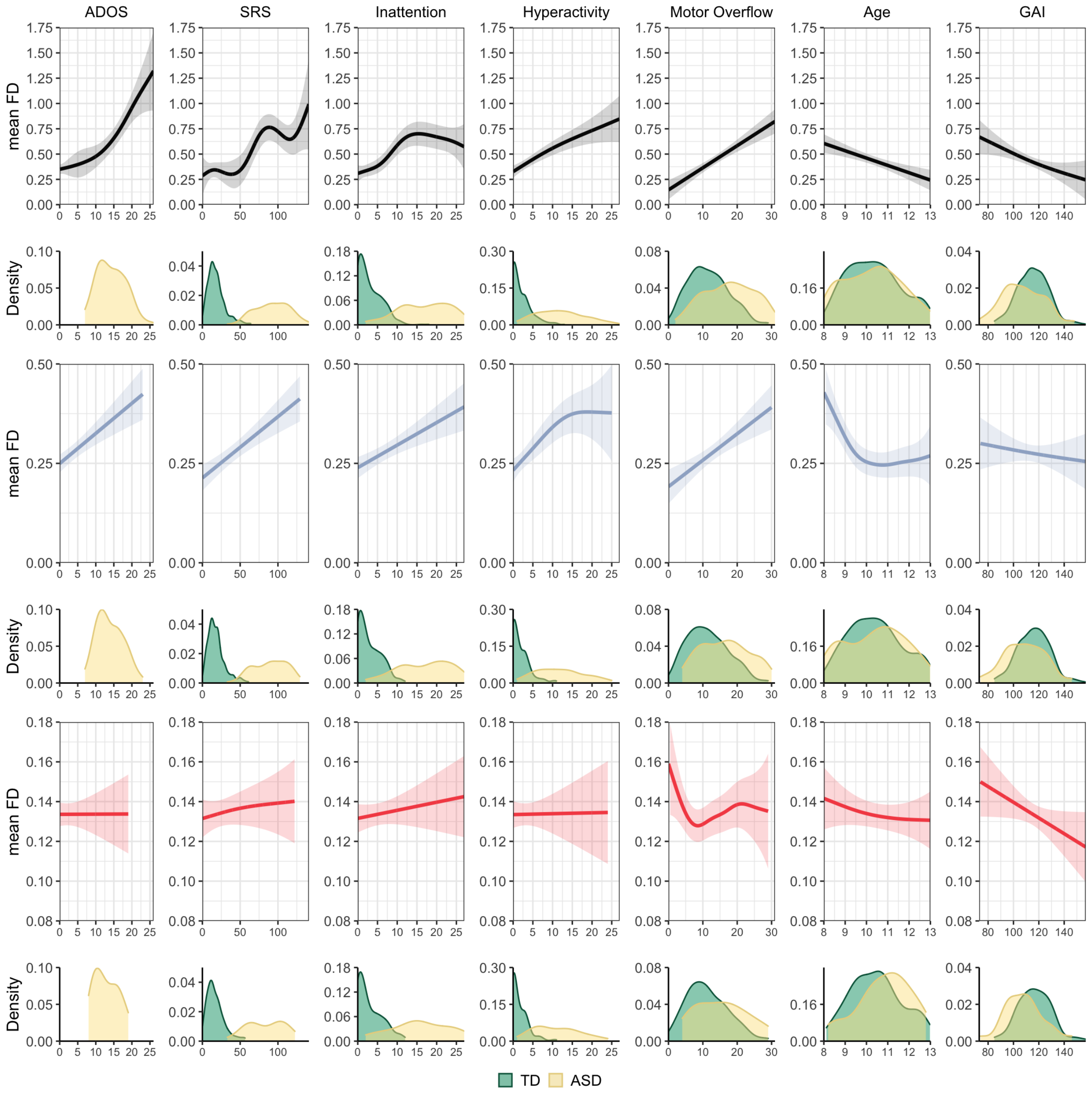

Figure S.2: **Univariate analysis of mean framewise displacement as a function of participant characteristics**. From left to right: Autism Diagnostic Observation Schedule (ADOS) total scores, social responsiveness scale (SRS) scores, inattentive symptoms, hyperactive/impulsive symptoms, total motor overflow, age, and general ability index (GAI) for children with usable and unusable rsfMRI data (top row, black lines), children with usable data under lenient motion QC (slate blue lines), and children with usable data under strict motion QC (red lines). Variable distributions for each diagnosis group are displayed across the bottom panel (TD=typically developing, green; ASD=autism spectrum disorder, yellow).

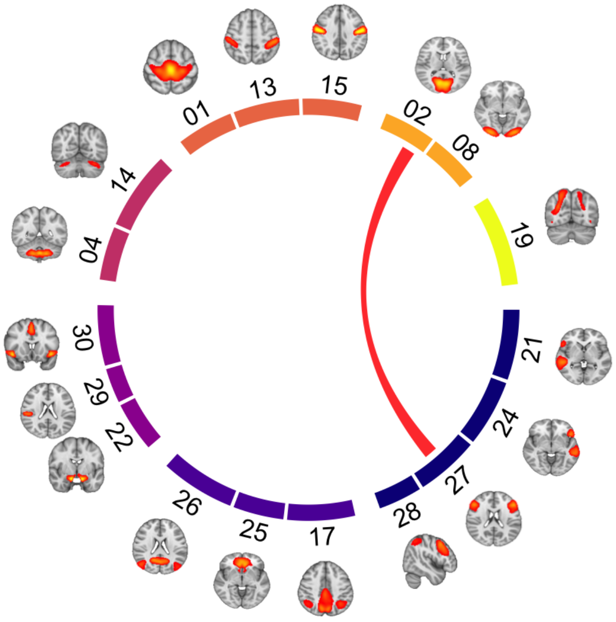

(a) Naïve Z-Statistic

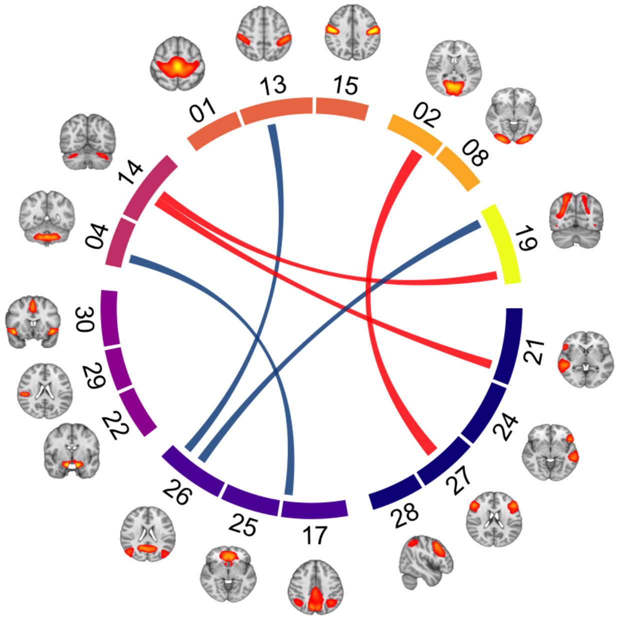

(b) DRTMLE Z-Statistic

Figure S.3: **Comparison of edges showing a group difference using the naïve and DRTMLE approaches at FDR=0.05**. Z-statistics for autism spectrum disorder (ASD) versus typically developing (TD) using A) the naive test and B) the deconfounded group difference using DRTMLE. Connections are thresholded at FDR=0.05. Blue lines indicates ASD>TD (0 in naïve, 3 in DRTMLE). Red lines indicates ASD<TD (1 in naïve, 3 in DRTMLE). Brain regions contributing to each independent component are illustrated and components are grouped by functional assignment. Navy nodes: control. Blue violet: default mode. Purple: salience/ventral attention. Magenta: pontomedullary/cerebellar. Coral: somatomotor. Orange: visual. Yellow: dorsal attention.

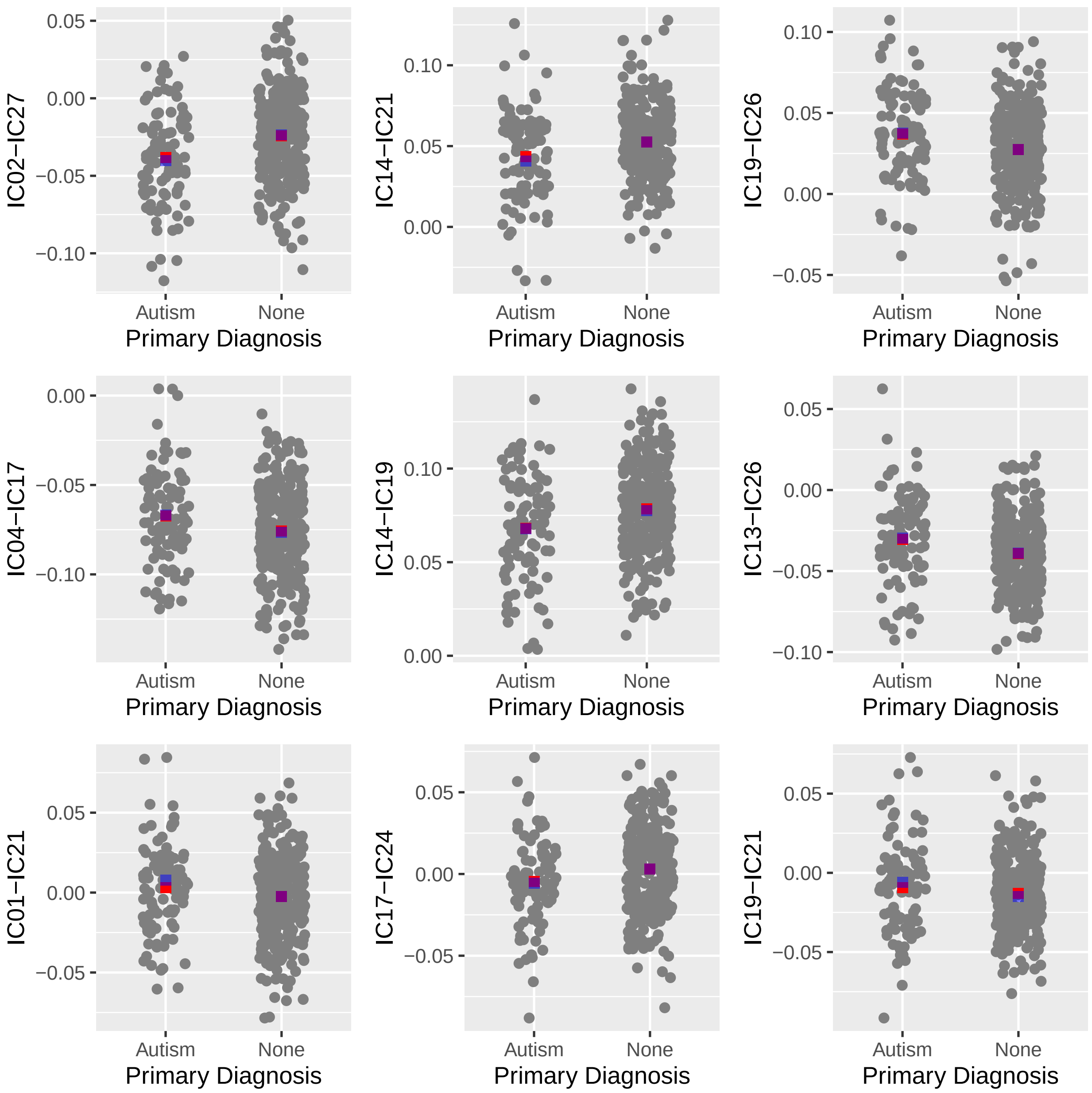

Figure S.4: **Plots of partial correlations, naïve means, and DRTMLE means for each diagnosis group for the nine components with the smallest DRTMLE p-values**. Naïve means appear in red. DRTMLE means appear in blue, with opacity such that means close to each other result in purple. Partial correlations are equal to the adjusted residuals described in the main manuscript Section 2.3.3. These plots indicate that there is large within group variability in the partial correlations. When applied in conjunction with the lenient motion QC, the DRTMLE means are very similar to the naïve means.
